# Free Volume Effect via Various Chemical Structured Monomers on Adhesion Property and Relative Permittivity in Acrylic Pressure Sensitive Adhesives

**DOI:** 10.3390/polym12112633

**Published:** 2020-11-10

**Authors:** Jung-Hun Lee, Ji-Soo Kim, Hyun-Joong Kim, Kyujong Park, Jungwoo Moon, Jinyoung Lee, Youngju Park

**Affiliations:** 1 Carbon Composite Materials Research Center, Institute of Advanced Composite Materials, Korea Institute of Science and Technology, Jeonbuk 55324, Korea; junghunlee@kist.re.kr; 2 Lab. of Adhesion and Bio-Composites, Program in Environmental Materials Science, Department of Agriculture, Forestry and Bioresources Seoul National University, Seoul 08826, Korea; kimjisoo2467@snu.ac.kr; 3 Research Institute of Agriculture and Life Sciences, College of Agriculture and Life Sciences, Seoul National University, Seoul 08826, Korea; 4 Samsung Display Co., Ltd., Yongin 17113, Korea; kj98.park@samsung.com (K.P.); jw75.moon@samsung.com (J.M.); ponchon.lee@samsung.com (J.L.); yjustin.park@samsung.com (Y.P.)

**Keywords:** acrylic pressure-sensitive adhesive, monomer, adhesion performance, relative permittivity, free volume

## Abstract

Acrylic pressure-sensitive adhesives (PSAs) are used as fixatives between layers of a display. PSAs’ function is an important factor that determines the performance of the display. Of the various display types available, the touch screen panel (TSP) of smart devices is firmly related to the relative permittivity of the elementals. Therefore, adjusting the relative permittivity of the PSA is indispensable for driving the TSP. Accordingly, selected acrylic pre-polymers were polymerized and the pre-polymer was blended and cross-linked with monomers with different chemical structure to adjust the relative permittivity. The monomers were hexametyldisiloxane (HMDS), N-vinylcaprolactam (NVC), tert-butyl acrylate (TBA), and isooctadecyl acrylate (ISTA). The gel fraction and transmittance as a function of the monomers show a similar result to the pure acrylic PSA. However, the gel fraction value decreased to about 90% and the transmittance decreased to about 85%, due to the immiscibility between nonpolar HMDS and acrylic PSA. On the other hand, the adhesion properties were improved when NVC was added because of the polarity of the nitrogen group. In addition, the relative permittivity of the PSA decreased regardless of the monomer chosen. There was, however, a difference in the optimal content of each monomer, and NVC decreased from 4 phr content to about 3.4 in reducing relative permittivity. Through the above results, it was confirmed that NVC having a nitrogen group is most advantageous in lowering adhesion properties and relative permittivity, and necessitates further research based on the findings.

## 1. Introduction

Pressure-sensitive adhesives (PSAs) are widely used in various industrial fields, such as medical products, aircraft, space shuttles, electrical devices, optical products, and automobiles. In particular, the demand for functional PSAs is rapidly increasing in the display market [1]. Displays are composed of a plurality of layer types and PSAs play an important fixative role. The PSA can fix the base material by applying slight pressure and becomes easy to adjust, while the processing rate can be increased [2]. Recently, to improve the technological development of displays, broader properties of the PSA function are required including transparency high elongation, high recovery, and heat dissipation [3].

Optical clarity means a transmittance of 90% or more in the range of 400 to 800 nm and PSA satisfies this criteria defining the utility of optical clear adhesives (OCAs). PSA includes rubber, acrylic, silicone, and urethane, and acrylic PSA is widely used in terms of their optical clarity. Moreover, the process time can be significantly reduced, and UV crosslinking is considerably economical as a technique. As the transmittance of PSA is a very important factor in display quality, a low transmittance may limit its application in the display industry [4,5].

**Figure 1:**
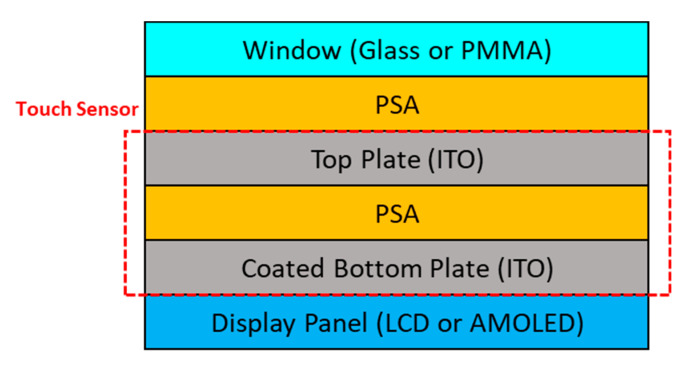
Structure of capacitance type touch screen panel (TSP). PSA, pressure-sensitive adhesive; ITO, indium tin oxide.

A touch screen panel (TSP) is a major technology for increasing input convenience and reducing product weight by replacing the keyboard in the display of electronic devices. The demand for TSP has soared owing to the spread of smart phones and tablet PCs, and it is essential to improve touch sensitivity. A capacitive touch screen panel is composed of a top and bottom plate, on which a transparent electrode (indium tin oxide, ITO) is deposited and each layer is fixed using PSAs (see Figure 1). As part of the capacitive method, a current flow operates through ITO on one side of the glass or a transparent film and a touch operation is recognized by sensing a change in capacitance that occurs when a finger or a touch pen touches the touch screen surface.

**Figure 2:**
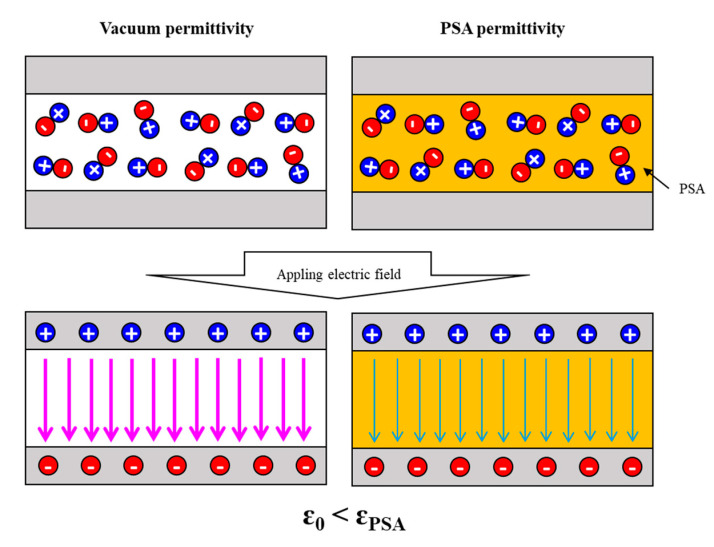
Schematic of polarization of electric charges and permittivity in vacuum and PSA.

The relative permittivity is a measure of the degree of polarization of electric charges inside when an electric field is applied to the non-conductor surface from the outside. From this point of view, the non-conductor is called the dielectric (ε). The relative permittivity (ɛ_γ_, dielectric constant) refers to the ratio of the permittivity of the material base relative to the permittivity in a vacuum. In other words, the relative permittivity is determined by the permittivity of a substance (see Figure 2) [6] and can be expressed by the following equation:

ɛ_γ_ = ɛ/ɛ_0_ (1)

Over the past decade, the performance of microelectronic integrated circuits (ICs) that make-up electronic devices has been improved owing to an increase in transistor speed and a decrease in size. ICs have become faster and more complex because smaller transistors run faster. However, these efforts are causing a decrease in the speed of signal propagation. The signal delay can increase with the size of the electronic device, which can degrade the performance of the final product. In other words, continuous shrinkage increases the speed of the transistor, but decreases the interconnection between the transistors. A way to improve this is to use a non-conductor with a low dielectric constant [7]. Non-conductors with a relative permittivity of 4.2 or less are called low-*k* dielectrics [8]. In addition, the relative permittivity of the capacitive TSP is closely related to the touch sensitivity. In order for PSA to be applied to TSPs, the relative permittivity of the material that affects touch sensitivity becomes a very important factor. If the permittivity is not adjusted, noise may be easily detected or signal transmission time may be delayed [9]. Therefore, the factor controlling the relative permittivity of the dielectric itself is very important. The relative permittivity control method includes a method of lowering the polarity using polarizable groups such as C-H, C-C, C-O, C-Si, and C-F bonds and a method of lowering the density by introducing free volume and nano-pores [10,11,12,13,14,15,16,17,18,19,20,21,22,23]. However, research results have also been reported that such voids can affect mechanical properties [24,25,26,27,28,29].

In this research, the possibility of adjusting the relative permittivity of monomers composed of various chemical structures was analyzed and mapped to behavioral changes in adhesion properties. An acrylic pre-polymer was synthesized via UV polymerization with acrylate monomers. In addition, four types of monomers were chosen and mixed with the pre-polymer. An acrylic PSA sheet was prepared via photo-crosslinking as a function of the monomer content. The crosslinking degree of the PSAs was confirmed via gel fraction and the transmittance was measured using a UV/vis spectrophotometer. The adhesion property was measured via the peel strength, probe tack, and lap shear strength. The relative permittivity was measured using a micro vacuum probe station to obtain reliable data as a function of chemical structure and content of the monomer. Through these results, we tried to confirm the applicability of the low-*k* monomer in controlling the relative permittivity, adhesion performance, and optical property of the acrylic PSA for TSP. In addition, it was attempted to secure a reliable relative permittivity value of semi-solid samples at room temperature as a result of a low glass transition temperature (T_g_).

## 2. Experimental Section

### 2.1. Materials and Chemicals

**Figure 3:**
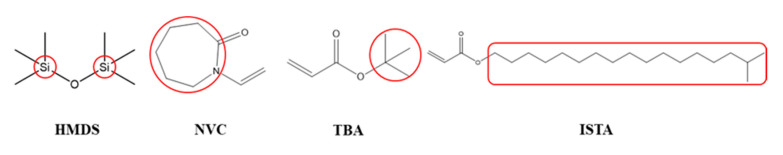
Chemical structure of monomers. HMDS, hexamethyldisiloxane; NVC, N-vinylcaprolactam; TBA, tert-butyl acrylate; ISTA, isooctadecyl acrylate.

Reactive acrylic monomers, 2-hydroxyethyl acrylate (2-HEA, 99.0%), 2-ethylhexyl acrylate (2-EHA, 99.0%), and isobutyl acrylate (IBA, 99.0%), were purchased from Samchun Pure Chemical (Republic of Korea). They were used as received without further purification to synthesize the acrylic pre-polymers. Hexamethyldisiloxane (HMDS, Sigma-Aldrich, St. Louis, MO, USA), N-vinylcaprolactam (NVC, Tokyo Chemical Industry Co., Ltd., Tokyo, Japan), tert-butyl acrylate (TBA, Sigma-Aldrich, USA), and isooctadecyl acrylate (ISTA, Nippon Shokubai Co., Ltd., Osaka, Japan) were selected as the monomers to adjust relative permittivity of the acrylic PSA via introduction of free volume (see Figure 3). 2-hydroxy-2-methyl-1-phenyl-1-one (Irgacure 1173, BASF, Ludwigshafen, Germany) and Phosphine oxide (Irgacure 2100, BASF, Germany) were used as the photoinitiator for the acrylic pre-polymer synthesis and UV crosslinking, respectively. Crosslinking for the pre-polymer was used 1,6-hexanediol diacrylate (HDDA, Sigma-Aldrich, USA).

### 2.2. Acrylic Pre-Polymer Synthesis

The acrylic monomers were blended with an Irgacure 1173 inside a 500 mL four-neck round-bottomed flask equipped with a stirrer, thermometer, and N_2_ purging tube (the formulation of the synthesized acrylic pre-polymer is listed in Table 1). The mixture was continuously stirred for 20 min at room temperature with N_2_ gas. The monomer mixture was synthesized by UV irradiating using a UV-spot cure system (SP-9, USHIO, Tokyo, Japan) under a N_2_-rich atmosphere until the temperature of the mixture rose by 5 °C. The above process was iterated five times and the product was stored in a wide-mouth bottle to protect the acrylic pre-polymer from light and air.

### 2.3. UV Crosslinking of Acrylic PSAs with Monomers

PSA sheets with monomers were prepared by blending 100 wt.% of the synthesized acrylic pre-polymer with 1 part per hundred resin (phr) of HDDA and Irgacure 2100 as a function of the monomer content (see Table 2). The mixture was combined and deformed using a paste mixer (SR-500, Thinky, Tokyo, Japan) for 4 min. The blends were coated onto the surface of corona-treated polyethylene terephthalate (PET) films about 50 μm in thickness and cross-linked by UV light, approximately 1300 mJ/cm^2^.

### 2.4. Characterizations

To confirm the influence of monomers on the crosslinking degree of the PSA, the gel fraction was measured as a function of the monomer content. The gel fractions of cross-linked PSA as a function of HDDA content were determined by soaking about 5 g of the PSA in toluene for 24 h at room temperature with shaking. The soluble part was removed by filtration and dried at 80 °C for 6 h to obtain a constant weight. The gel fraction was calculated by the following equation:

Gel fraction (%) = (W_1_/W_0_) × 100 (2)

where W_0_ and W_1_ are the weights before and after soaking and filtration, respectively.

Visible light transmittance of the specimens as a function of monomer type and content was measured in the wavelength of 400 to 800 nm using UV/vis spectrophotometry (Cary 100, Agilent Technologies, Santa Clara, CA, USA). The PSA specimens were prepared to a thickness of approximately 400 μm.

The peel strengths of the PSAs according to the type and content of monomer were investigated using a texture analyzer (TA-XT2i, Micro Stable Systems, Godalming, UK). Firstly, 25 mm width PSA films were attached to a stainless steel (SUS) substrate and pressed twice by a 2 kg rubber roller. The peel strength was determined at an angle of 180° with a crosshead speed of 300 mm/min at room temperature based on ASTM D3330. The specimens were pressed onto stainless steel (SUS) substrates by two passes of a 2 kg rubber roller and then stored at room temperature for 24 h. Each sample was repeatedly measured three times, and the average value was calculated with N/25 mm.

Lap shear testing was investigated using a texture analyzer. The tested specimens were cut into smaller pieces with a width of 25 mm. After being removed from a silicone release film, each PSA film was attached to another PET substrate (the adhesion cross-sectional area was equal to 25 × 25 mm^2^ and a 2 kg rubber roller was passed over the film surface three times). The lap shear tests were performed at a crosshead rate of 5 mm/min. The shear strain rate values were calculated using the following equation:

Shear strain rate (%) = ΔL/t × 100(3)

where ΔL is the moving distance and t is the thickness of the PSA film.

The shear stress property is one of the important factors of PSAs for display applications. Therefore, researching the relationship between the shear strain rate and the thickness of the applied PSA film is imperative for future use in the display industry [30].

**Figure 4:**
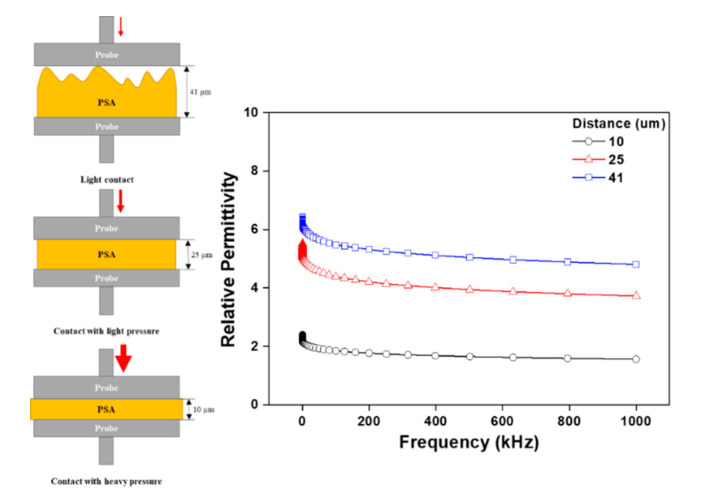
Difference in relative permittivity results using a surface/surface probe as a function of PSA distance.

**Figure 5:**
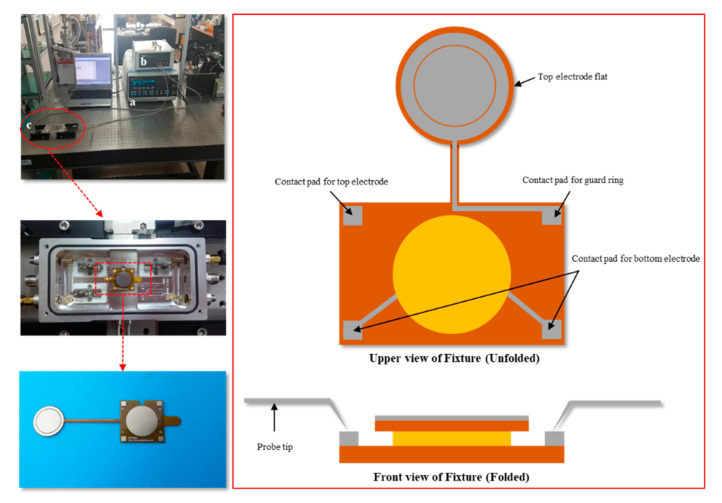
Equipment and fixture scheme to measure the relative permittivity for the PSA films: (a) impedance analyzer, (b) dielectric interface, and (c) micro vacuum probe station.

Figure 4 is an evaluation of the reliability of the relative permittivity data of PSAs using a surface/surface probe. Although general polymers have very high data reliability, the nature of PSA is semi-solid and the thickness of the specimen is sensitively changed by the pressure of the surface/surface probe. General polymers have very high data reliability, but the semi-solid nature of the PSA against the thickness of the specimen may interfere with the sensitively of the touch pressure at the surface/surface probe interface. Hence, for this reason, the reproducibility of the data was a concern and it was difficult to establish a reliable correlation. Figure 5 shows the analysis equipment for obtaining data with a higher reliability of PSA. It consisted of (a) an impedance analyzer (1260 Impedance Analyser, Solartron Analytical, UK), (b) a dielectric interface (1296 Dielectric Interface, Solartron Analytical, UK), and (c) a chamber (micro vacuum probe station, NEXTRON Co., Ltd., Republic of Korea). The permittivity of a PSA sheet was investigated using the more delicate micro vacuum probe station. The frequency range was set from 1 to 1000 kHz and the thickness of all PSA films was approximately 400 μm with the sample diameter detector fixed at a circular cross-section of 10 mm.

## 3. Results and Discussion

### 3.1. Gel Fraction

**Figure 6:**
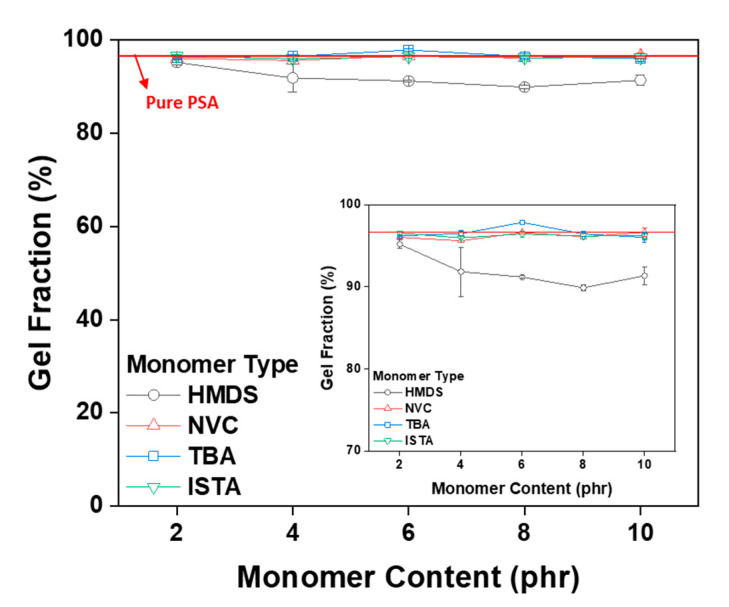
Gel fractions of crosslinked acrylic PSAs as a function of monomer type and contents.

The cross-linking degree of the acrylic PSA was indirectly confirmed by calculating the insoluble portion in toluene. Figure 6 is a graph of the gel fraction results according to the type and content of monomer. The gel fraction value of the cross-linked PSAs decreased up to 90% as the HMDS content increased. This result is thought to be due to the lack of functional groups capable of reacting with the acrylic pre-polymer and the low interfacial surface energy between the acrylic-silane groups [31]. The silane group HMDS was selected because it is reported to be excellent in reducing the relative permittivity. The effect of the result on adhesion will be described in another session. It was confirmed, however, that the gel fraction value (in accordance with the content and all monomers except HMDS) was about 97% or more and did not inhibit the degree of crosslinking of the acrylic PSA. This result is attributed to the monomer C=C bond participation in the crosslinking process, preventing gel disintegration via bond weakening.

### 3.2. Transmittance

**Figure 7:**
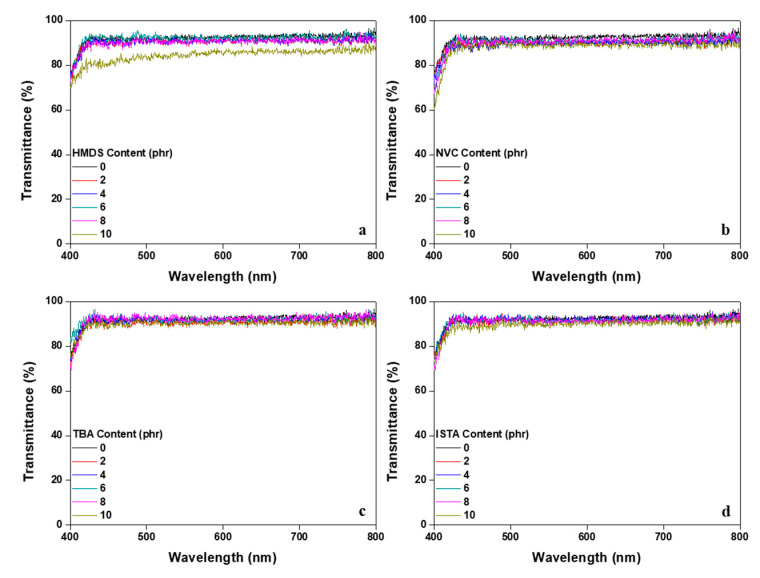
Transmittance from a UV/vis spectrophotometer of a crosslinked acrylic PSA as a function of monomer type and content: (**a**) PSA-HMDS; (**b**) PSA-NVC; (**c**) PSA-TBA; (**d**) PSA-ISTA.

Figure 7 is the measurement data of the transmittance in the visible light region as a function of monomer content type. Transmittance of the PSA is one of the important properties because it is closely related to the image quality of the display. Irrespective of the content type, the transmittance of all monomers was similar to that of the neat acrylic PSA, with the exception of HMDS. In the case of HMDS, however, comprising the same silane group, it was confirmed that the transmittance was not significantly different from the gel fraction result, but was observed to decrease to about 85% of the original size at 10 phr content. It was noted that the transparency of the acrylic PSA decreased as a result of immiscibility between the acrylate and silane group in HMDS. This problem was deemed to be limited to transparent display applications and largely dependent on the application site.

### 3.3. Adhesion Performances

**Figure 8:**
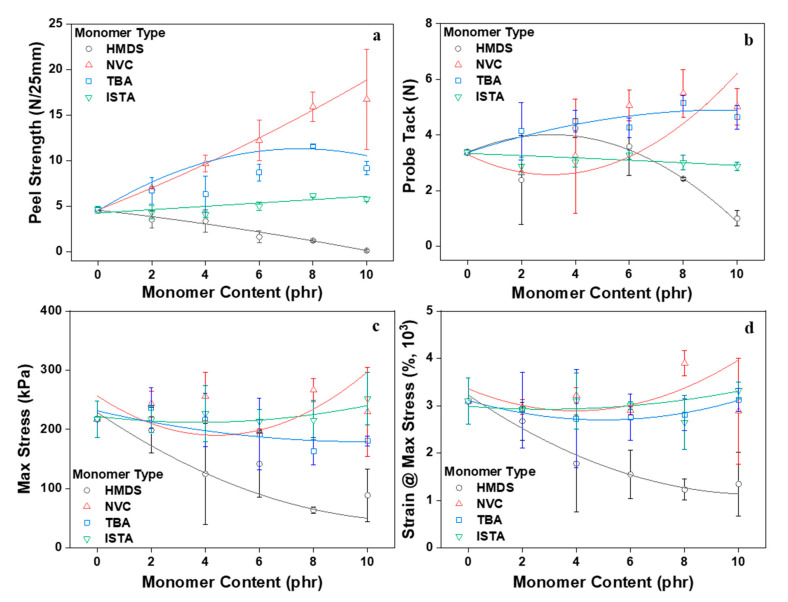
Adhesion performances of PSAs as a function of monomer type and contents: (**a**) peel strength; (**b**) probe tack; (**c**,**d**) maximum stress and strain at maximum stress from lap shear test.

**Figure 9:**
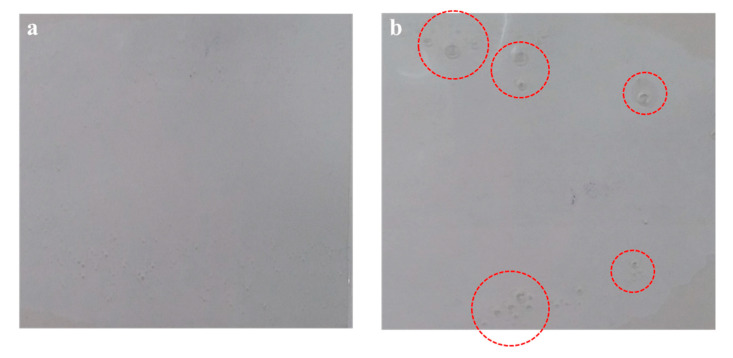
Pictures of crosslinked acrylic PSA films: (**a**) pure acrylic PSA and (**b**) PSA-HMDS (10 phr).

The underlying behavior of PSAs is related to adhesion, cohesion, and tack-related performance. Adhesion refers to adhesion to the substrate, cohesion refers to internal bonding strength, and tack refers to contact adhesion to the substrate [2]. Thus, in order to measure the adhesion properties of the PSA mapped by the content type of the monomer composition, peel strength, probe tack, and lap shear tests were conducted (see Figure 8). The peel strength and the probe tack value of HMDS with increasing PSA showed a decreasing trend with HMDS owing to the silane group content. In lap shear test results, maximum stress and strain at maximum stress decreased with increasing HMDS content. In general with PSAs, when a network is formed by crosslinking, the cohesion force increases and the tacky and peel strength values of the PSA decrease [32,33,34,35]. However, in the case of HMDS, as a result of measuring the gel fraction, the degree of crosslinking decreased, and the adhesion properties decreased as the content increased. In addition, one of the disadvantages of general acrylic PSAs is that it has very low adhesion properties relative to low surface energy substrates such as polyethylene (PE), polypropylene (PP), and polydimethylsiloxane (PDMS). For this reason, a silicone-coated PET film is used as a protective film. Unlike other monomers, HMDS has a low polarity, so its adhesion properties to a substrate with low surface energy are very low. That is, the attraction between the molecular chains of the acrylic PSAs and the HMDS is weak, and this also contributes to lowering the adhesion properties of the PSAs. The peel strength of PSAs increased consistently with the NVC content as a result of the improvement in the intermolecular interaction with a relatively high polarity caused by the presence of the NVC nitrogen atom. Its inclusion played a role in improving the cohesion of the acrylic PSA [36,37]. For this reason, probe tack values and lap shear results also increased with the increasing content. However, the effect of polarity had relatively little effect on the shear direction. On PSA with TBA and ISTA, peel strength slightly increased as the content increased with no significant difference. When comparing TBA and ISTA in terms of chemical structure, ISTA with a comparatively larger molecular weight decreased the wettability and its presence was marked by an overall decrease compared with the branch structured TBA configuration. Probe tack results also showed a similar tendency with NVC as the TBA content increased, whereas ISTA having a long alkyl chain group did not significantly affect the value. Similar to NVC, TBA and ISTA also did not have a significant impact on lap shear values. However, it should be noted that the value was higher than that of the PSA to which HMDS was added, and the standard deviation accordingly decreased. This result shows a more stable result because NVC, TBA, and ISTA have C=C groups capable of reacting with residual acrylic monomers, crosslinking agent, and acrylic pre-polymers unlike HMDS. On the other hand, the HMDS monomer that did not participate in the reaction instead generated a bubble-like content during the crosslinking process of the acrylic PSA. The resulting phenomenon is ascribed to the adhesion strength decrease accompanied by an increase in the standard deviation (see Figure 9).

### 3.4. Relative Permittivity

**Figure 10:**
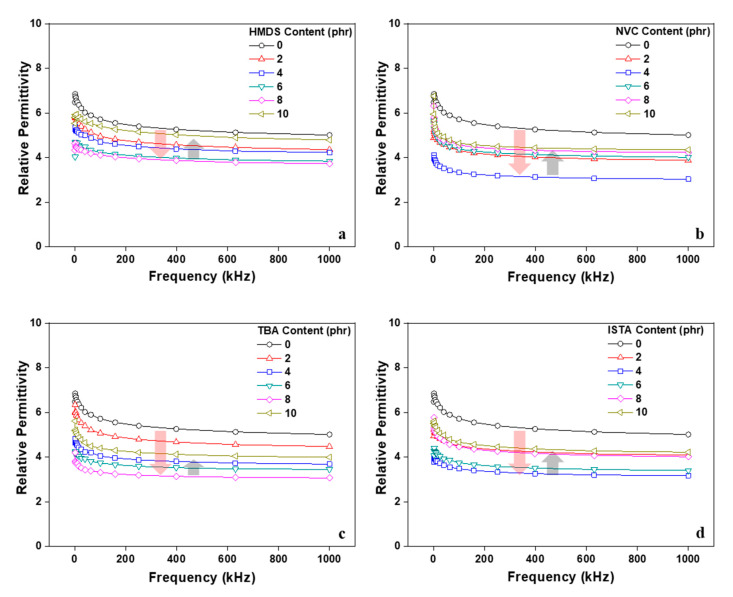
Relative permittivity of cross-linked acrylic PSAs as a function of monomer type and contents: (**a**) HMDS; (**b**) NVC; (**c**) TBA; and (**d**) ISTA.

**Figure 11:**
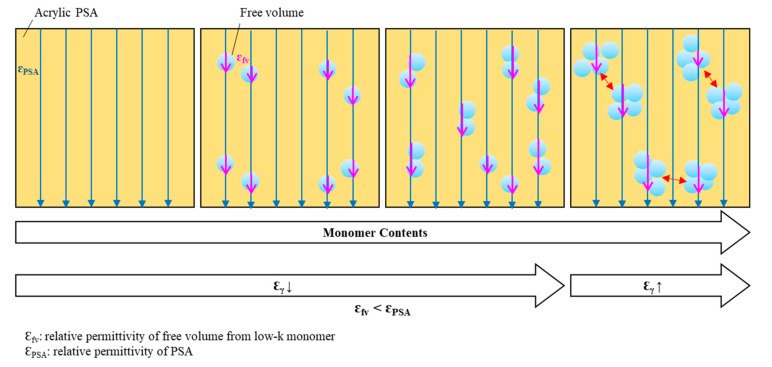
Schematic of free volume distribution in acrylic PSAs as a function of monomer contents.

The relative permittivity in the low frequency region according to the type and content of the monomer was investigated as shown in Figure 10. Regardless of the chemical structure of monomer, the relative permittivity of the acrylic PSA to which the monomer was added began to decrease in all measured frequency ranges investigated. This may arise as a result of the participation of the monomer in a crosslinking reaction in the acrylic pre-polymer resulting in a form a free volume between the acrylic molecular chains [24,25,26,27,28,29]. However, the relative dielectric constant of the acrylic PSA was observed to rise gradually above a certain threshold of the monomer content. The threshold marked differences that related to the functional groups of the monomer. HMDS and TBA began to increase at 8 phr or more, and NVC and ISTA at 4 phr or more. This result can be explained in two ways. Firstly, the monomer (especially HMDS) that did not react with the acrylic pre-polymer was not present in the acrylic PSA, as shown in the gel fraction and adhesion performances. Secondly, when the content of the monomer exceeds a certain level, it becomes excessive compared with the capacity and monomer aggregation results in a decrease in the efficiency in the free volume formation. Through this result, the monomers were effective in lowering the relative permittivity of the acrylic PSAs (see Figure 11). In addition, it was confirmed that there is a difference in the optimal content according to the chemical structure of the monomers.

**Figure 12:**
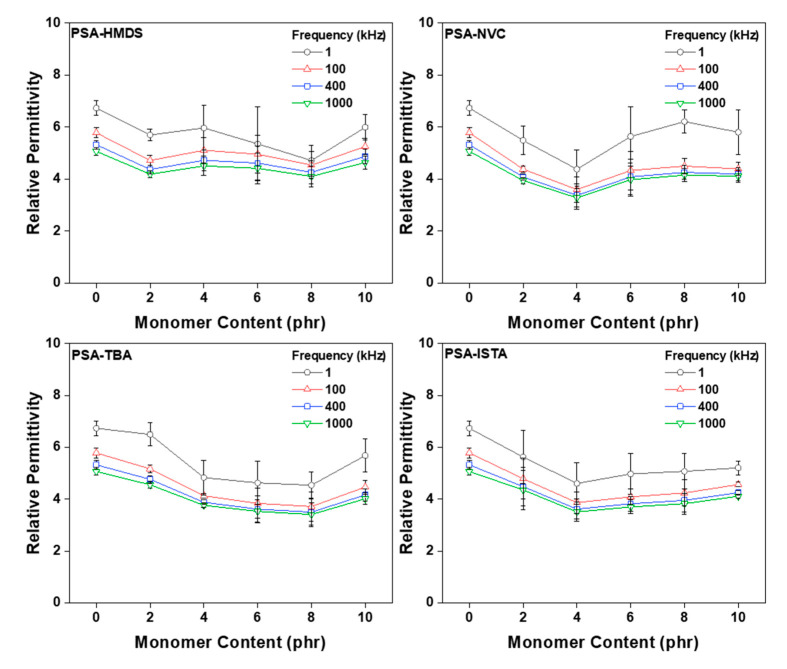
Relative permittivity at specific frequency (1, 100, 200, 400, 1000) of cross-linked acrylic PSA as a function of monomer type and contents.

In the PSA display, the value of the relative permittivity at a particular frequency is very important. In particular, the relative permittivity that accompanies the touch sensitivity of the TSP is related to a low frequency region. In this region, a degree of variability in the relative permittivity is expected and deviation from the required value varies with frequency. Figure 12 is a graph that summarizes the relative permittivity at a specific frequency. As mentioned above, the relative permittivity was observed to decrease upon addition of the monomer and this tendency varied depending on the chemical structure of the monomer. As in the lap shear test result, it is believed that air bubbles may occur by evaporation of the aggregated monomers from heat generation via ultraviolet exposure during the crosslinking process. In addition, the air bubbles had a greater effect on the relative permittivity than the adhesion performances. According to previous studies, it has been reported that the relative permittivity of polymer can vary greatly depending on the air gap [38,39,40].

## 4. Conclusions

A study was conducted on the relative permittivity of PSA, which is closely related to the touch sensitivity of TSP. To lower the relative permittivity of PSA, four types of monomers were selected. The gel fraction value and transmittance according to the chemical structure and content of the monomer did not show a significant difference compared with the pristine acrylic PSA. However, at 10 phr of HMDS comprising a silane group, the gel fraction value decreased to about 90% and the transmittance decreased to about 85%. As a result, the adhesion performances deteriorated owing to immiscibility between the nonpolar HMDS and the acrylic molecular chain. In contrast, the adhesion properties improved as a result of the polarity reaction by the nitrogen group of NVC, TBA, and ISTA. Branched and long alkyl chain grouped monomers did not significantly affect the adhesion properties. In addition, it was confirmed that the monomers devoid of functional groups capable of reacting with the acrylic pre-polymer remained generating bubbles by heat evaporation and impacting the adhesion properties. Regardless of the type of monomer, the relative dielectric constant of the PSA decreased and the relative permittivity began to rise above a certain content. This is because of the generation of air bubbles due to heat generation in the cross-linking process, likely resulting from monomer aggregation. In hindsight, the optimum content of the monomer was effective in lowering the relative permittivity as a result of the formation of free volume, but the adhesion performances and the relative dielectric constant were affected by bubbles caused by evaporation.

As shown in this study, the adhesion performance changed according to the type of monomer, and as a result, the relative permittivity can be adjusted according to monomer content. In the future, we plan to further investigate the application methods for monomer control, adhesion properties, and relative permittivity of acrylic PSA. We aim to study the behavior of adhesion performance and relative permittivity when the dispersibility of monomer is enhanced by producing a pre-polymer by simultaneously reacting reactive acrylic monomer and monomer via in situ polymerization.

## Figures and Tables

**Table 1:** Formulation of acrylic pre-polymer. 2-HEA, 2-hydroxyethyl acrylate; 2-EHA, 2-ethylhexyl acrylate; IBA, isobutyl acrylate.

Sample Names	Reactive Monomers		
	2-HEA (wt.%)	2-EHA (wt.%)	IBA (wt.%)
Acrylic Pre-Polymer	20	60	20

Photoinitiator: 2-hydroxy-2-methyl-1-phenyl-1-one, Irgacure 1173.

**Table 2:** Formulation of cross-linked acrylic pre-polymer/monomer blends. PSA, pressure-sensitive adhesive; HMDS, hexamethyldisiloxane; NVC, N-vinylcaprolactam; TBA, tert-butyl acrylate; ISTA, isooctadecyl acrylate.

Sample Names	Acrylic Pre-Polymer (wt.%)	Monomers			
		HMDS (phr)	NVC (phr)	TBA (phr)	ISTA (phr)
PSA-HMDS	100	2/4/6/8/10	-	-	-
PSA-NVC		-	2/4/6/8/10	-	-
PSA-TBA		-	-	2/4/6/8/10	-
PSA-ISTA		-	-	-	2/4/6/8/10

Crosslinking agent: 1,6-hexanediol diacrylate, HDDA; Photoinitiator: phosphine oxide, Irgacure 2100.
